# Results of calcanectomy in the surgical management of heel ulceration with osteomyelitis in the high risk patient

**DOI:** 10.1186/1757-1146-3-S1-O28

**Published:** 2010-12-20

**Authors:** Ben Yates

**Affiliations:** 1 Great Western Hospital, Swindon, UK

## Introduction

Heel ulceration associated with calcaneal osteomyelitis is a very difficult pathology to treat and often results in below knee amputation. Surgical excision of the infected bone and ulcer via calcanectomy has been shown to reduce amputation and mortality rates. The purpose of the study was to review our experience with this procedure and examine factors that might influence outcome.

## Method

Between 2005 and 2010 sixteen patients underwent partial or complete calcanectomy for chronic heel ulceration associated with osteomyelitis. The case notes were reviewed to determine; outcome, pre-operative size and depth of ulceration, degree and type of bone involvement, cause of neuropathy, peripheral vascular disease, BMI, nutritional status, infective organism based upon bone cultures.

## Results

Eleven of the sixteen patients had diabetes. All had neuropathy. Thirteen of the sixteen wounds healed without the need for below knee amputation at a mean of 8 weeks (range 3-28). Factors that could influence outcome in the three failures included; diabetes, pathological fracture involving the subtalar joint, infective organism. The size of the ulcer, BMI, presence of PVD and nutritional status were not relevant factors.

## Conclusion

Calcanectomy is an effective alternative to below knee amputation for chronic heel ulceration with bone infection.

